# Assessing the Variations in Breast/Ovarian Cancer Risk for Chinese *BRCA1/2* Carriers

**DOI:** 10.1155/2022/9390539

**Published:** 2022-03-26

**Authors:** Ang Li, Shuai Hao, Jiaqi Luo, Yi Zi, Zhaoji Lan, Tianliangwen Zhou, Qihuan Zhi, Jiamin Zhan, Gang Sun, Yujian Shi, Donglin Luo

**Affiliations:** ^1^Department of Research, Top Gene Tech (Guangzhou) Co., Ltd., Guangzhou, China; ^2^Department of Breast, Thyroid Surgery, Daping Hospital, Army Medical University, Chongqing 400042, China; ^3^Department of General Surgery, Jinling Hospital, Medical School of Nanjing University, Nanjing 210002, China

## Abstract

**Background:**

Cancer risks vary in different *BRCA1/2* mutations. We are interested in identifying regions associated with elevated/reduced risks of breast/ovarian cancers in the Chinese population and comparing with previously reported Caucasian-based breast/ovarian cancer cluster regions (OCCR/BCCR). We also aim to characterize the distribution and estimate the cancer risks of different Chinese recurrent mutations.

**Methods:**

A total of 3,641 cancer-free women and 4,278 female cancer patients were included in the study. Germline *BRCA1/2* status was detected with amplicon-based next-generation sequencing. We calculated the odds ratio (OR) of breast cancer and OR of ovarian cancer, and their ratio of the two ORs (ROR) for each region. ROR >1 indicated elevated odds of breast cancer and/or decreasing odds of ovarian cancer, and vice versa. The frequency, distribution, and penetrance of six known Chinese founder mutations were characterized, respectively. Haplotype analysis and age estimation were performed on the most prevalent founder mutation *BRCA1*: c.5470_5477del.

**Results:**

A total of 729 subjects were detected with germline *BRCA1/2* deleterious mutations. The putative Chinese OCCR/BCCR partially overlapped with Caucasian-based OCCR/BCCR and shared structural-functional characteristics. The six known Chinese founder mutations greatly vary in both distribution and penetrance. The two widely spread mutations are estimated to convey low penetrance, while the area-restricted founder mutations seemed to confer higher/complete penetrance. *BRCA1*: c.5470_5477del is estimated to have emerged ∼2,090 years ago (70 B.C.) during the Han dynasty.

**Conclusions:**

*BRCA1/2* carriers with different genotypes have significantly different cancer risks. An optimal risk assessment should be mutation specific, rather than concerning a single figure.

## 1. Introduction

Since the establishment of predisposing effects of *BRCA1* (MIM:113705) and *BRCA2* (MIM: 114480) to breast/ovarian cancers, we have accumulated understandings of the biological roles of *BRCA1/2* and their prevalence of mutations in different ethnic populations through a lot of functional experiments and sample-based studies. It is now generally believed that deleterious mutations in *BRCA1/2* or other related genes result in the production of malformed proteins that are unable to function during the error-free DNA repair process mediated by homologous recombination (HR) upon DNA double-strand break, which will lead to genomic instabilities and eventually the development of malignancies. It is also known that race/ethnic differences are presented in the mutation spectrum, the prevalence of mutations, and in the recurrently mutated positions, which are likely to reflect the so-called “founder effects.” For example, Ashkenazi Jews generally have a higher risk of being *BRCA1/2* carriers because of the highly prevalent founder mutations *BRCA1* 185delAG*, BRCA1* 5382insC, and *BRCA2* 6174delT [1]. Several Chinese founder mutations have also been previously reported [2–6].

Moreover, it is recognized that great differences in cancer risks present within *BRCA1/2* mutation carriers, depending on the location and type of mutation they bear. According to previous observations, mutations within certain regions (defined as ovarian cancer cluster regions, OCCRs) are associated with higher ovarian cancer and/or lower breast cancer risks than other regions and certain regions (defined as breast cancer cluster regions, BCCRs) with higher breast cancer and/or lower ovarian cancer risks. The biological impact caused by the mutation is the determining factor of cancer risk, which apparently affects the position of the OCCRs and BCCRs; the race/ethnicity of the studied population is another variable as it affects the mutation spectrum (e.g., the position of mutation hotspots). Several studies have calculated putative OCCR/BCCRs of *BRCA1/2* using samples mostly of Caucasian origin, and the estimated regions are considerably overlapping [7–9]. Interestingly, for both *BRCA1* and *BRCA2*, the estimated OCCRs seemed to locate in the center of the coding sequence (CDS) and significantly overlap with the largest exon (Exon10 of *BRCA1* and Exon11 of *BRCA2*), while the BCCRs seemed to occupy the 5' and the 3' ends of the CDS. As we expect race/ethnic difference to cause some degree of variability in OCCR/BCCRs when studying different populations, and since Asian populations only represented 1% of the total samples in previous studies, we are interested in defining OCCR/BCCRs in Chinese *BRCA1/2* carriers and finding out to what extent the position of OCCR/BCCRs could be varied. Moreover, it has been widely acknowledged that the identification and screening of founder mutations are highly cost-effective measures for cancer risk management [10]. Hence, we also aim to characterize the distribution of Chinese recurrent mutations (or founder mutations) and estimate the cancer risks they confer, in order to provide a reference to the precision management of genetic risks for Chinese *BRCA1/2* carriers.

## 2. Methods

### 2.1. Samples

A total of 3,641 cancer-free women and 4,278 female cancer patients (breast cancer, ovarian cancer, colon cancers, and pancreatic adenocarcinoma) in the nationwide of China volunteered to enroll in this study. The participants selected for further study should meet the following criteria: (1) ≥18 years old with unambiguous clinical status (cancer-free/cancer); (2) have taken the BRCA1/2 gene test; and (3) have unambiguous pathology and a sufficient amount of materials for the experiment if they were diagnosed with cancer. Clinical characteristics of samples in relation to *BRCA1/2* are presented in Table 1. Among the 3,641 cancer-free individuals, 2,615 were negative of family history and further selected as a healthy control for penetrance estimation and haplotype analysis. All the subjects involved had given their written informed consent in accordance with the Chinese ethical standards and the 2008 Helsinki Declaration.

### 2.2. *BRCA1/2* Genetic Testing and Mutation Classification

Blood samples were collected from all enrolled individuals and subjected to NGS-based *BRCA1/2* whole-exon sequencing (all coding regions and exon-intron boundaries20 bp) with average sequencing depth >1000x on the NextSeq CN500 platform (Berry Genomics, China). The analysis pipeline and mutation classification followed protocols as described in our previous research [11]. The human genome hg19/GRC37 was used as reference; the NCBI reference sequences NM_007294.3 and NM_000059.3 were used for annotations of *BRCA1* and *BRCA2* variants, respectively. Nomenclature of mutations followed the latest version of Human Genome Variation Society Sequence Variant Nomenclature (HGVS, https://varnomen.hgvs.org/) and Mutalyzer Name Checker (https://mutalyzer.nl). Deleterious mutations in this study include likely pathogenic and pathogenic mutations. Further validations of all deleterious mutations and variants of uncertain significance were performed by Sanger sequencing. Visualization of the variants was presented with circos plots [12].

### 2.3. Mutation Grouping and Statistical Analysis

To estimate OCCR/BCCR, segments of regions containing all deleterious mutations (regardless of mutation type or function) need to be created for statistical calculations. We divided the coding sequence (CDS) of *BRCA1* and *BRCA2* into bins by base pair location so that each bin contains a roughly equal number of carriers (Table 2). Large rearrangements were excluded to avoid spanning multiple bins.

We then calculate the odds ratio (OR) of breast cancer and the OR of ovarian cancer, respectively, for each bin. We computed a statistical measure ROR, defined as the ratio of breast versus ovarian cancer OR. The value of ROR is associated with elevated/reduced breast or ovarian cancer odds (ROR >1, increasing odds of breast cancer and/or decreasing odds of ovarian cancer; ROR <1, increasing odds of ovarian cancer and/or decreasing breast cancer odds). RORs of all bins were compared to identify significant outliers as putative OCCR/BCCRs. *P* values were adjusted with the Benjamini-Hochberg procedure to reduce the possibility of false positive. Adjusted *P* value <0.05 was considered significant.

### 2.4. Characterization of BRCA1: c.5470_5477delATTGGGCA

Among the recurrent mutations identified in this study, *BRCA1*: c.5470_5477delATTGGGCA occurred in 9.5% (50/527) of *BRCA1* carriers and had enough materials for further experimental validation. We then performed haplotype analysis on this variant. A total of 81 subjects (31 patients with the same mutations from independent families and 50 unrelated controls without the mutation) were included in the haplotype analysis. According to marker selection methods previously described [4, 13, 14], we selected nine polymorphic markers flanking the *BRCA1* gene-D17S800, D17S1320, D17S1321, D17S855, D17S1323, D17S1327, D17S1326, D17S1325, and D17S791 (short tandem repeats, STR, spanning approximately 5.8 Mbp, Supplementary Figure S1). We detected STR lengths using fluorescently end-labeled PCR primers and ABI 3730xl Genetic Analyzer (Applied Biosystems, Foster City, CA, USA). We reconstructed all possible haplotypes using PHASE v.2.1.1 [15] (Supplementary Table S1). A chi-square test was used to evaluate the differences in allele frequencies between patients and healthy subjects for each STR involved.

We also performed an age estimation of *BRCA1*: c.5470_5477delATTGGGCA. We used DMLE + v2.3 to estimate the original time when the mutation emerged in the *BRCA1* gene [16]. Based on a Markov Chain Monte Carlo algorithm, this program enabled Bayesian inference to estimate the age of the specific mutation with the knowledge of the patients' and controls' haplotypes or genotypes observed, physical distances between markers (cM), and the estimated population growth rate. The population statistics of ancient time points were taken from historical data [17]; the population figure of the year 1949 was collected from the National Bureau of Statistics of China (https://data.stats.gov.cn/index.htm). The equation (1) shown below was performed to calculate a list of average growth rates (‰ per year) from official population records [17] of different historical time points ranging from 684 B.C. to 1949 A.D (Table 3). The calculated growth rates slightly differ from each other, with a maximum of 7.81‰ (1741 A.D-present) and a minimum of 1.54‰ (2 A.D-present). For reference, the estimated growth rate of the year 2019 is 3.34‰ (https://data.stats.gov.cn/index.htm).

(1) $$\begin{array}{c} P_{1}\times {\left(1+X\right)}^{n}=P_{2}-P_{1},\;\text{i.e. }X=\sqrt[{n}]{\frac{P_{2}-P_{1}}{P_{1}}}\;-1, \end{array}$$

*X* = average growth rate, *n* = time till now (years), *P*_1_ = population as recorded *n* years ago, and *P*_2_ = Chinese population in 2020 = 1.4 billion.

### 2.5. Penetrance Estimations on Recurrent Mutations for Breast Cancer

**Figure 1:**
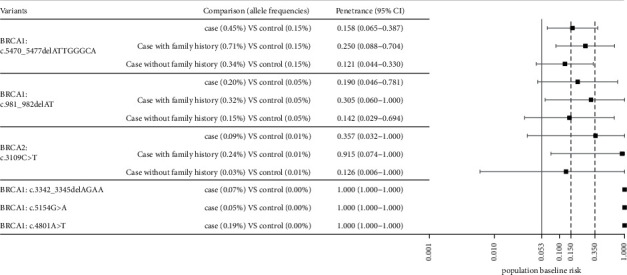
Penetrance estimations of six known Chinese founder mutations. The estimated population lifetime risk of breast cancer for Chinese women (derived from the Gail model) is 0.053; the estimated breast cancer risk in *BRCA1/2* carriers is 35–50% (lower bound: 0.35). Great variations in penetrance are observed among the six founder mutations.

We estimated the penetrance of the recurrent mutations of breast cancer using the allelic model by Bayes' theorem [18]. This formula required the knowledge of the lifetime risk of breast cancer in the Chinese population. Restricted by the access to the official data, we used the estimated mean value of lifetime risk (0.053; Figure 1, gray vertical line) for breast cancer derived from the Gail model among Chinese women [19] as the population baseline risk. The approximate lower bound (0.35; Figure 1, black dashed line) of breast/ovarian cancer risk in *BRCA1/2* carriers (according to previous studies [20, 21]) was used as a minimum expected penetrance of all deleterious mutations throughout the whole gene.

## 3. Results

Out of the total 7,919 samples containing 4,278 cancer patients and 3,641 cancer-free individuals, 729 were detected with *BRCA1/2* deleterious germline mutations, of which 539 individuals carried *BRCA1* mutations and 213 carried *BRCA2* mutations. The median age of breast cancer diagnosis was 43, 44, and 46 in *BRCA1* carriers, *BRCA2* carriers, and noncarriers; the median age of ovarian cancer diagnosis was 51, 54, and 52 in *BRCA1*, *BRCA2* carriers, and noncarriers, respectively.

**Figure 2:**
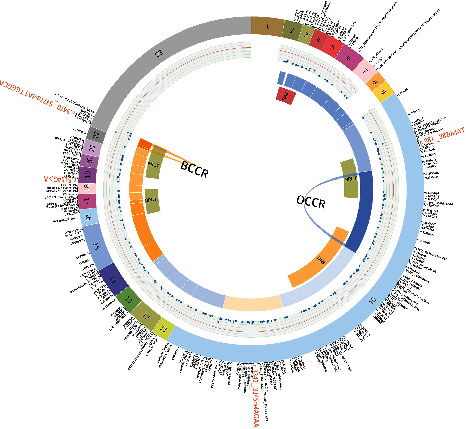
Circos plot representation of all *BRCA1* deleterious mutations identified in this study. The outmost ring displays the 23 exons of *BRCA1* and each variant at corresponding positions. Recurrent variants are highlighted in a red and enlarged font. The second circle is the mutation density graph, each dot corresponds to a variant, and its distance to the outmost ring represents the frequency (the closer the higher; the most prevalent mutation is colored in red). The ROR is represented by a heatmap in the intermediate ring. The next circle displays the *BRCA1* functional domains. The innermost arcs represent previously reported Caucasian-based OCCR/BCCRs. The areas enclosed by the arches indicate the estimated Chinese OCCR/BCCR, which are statistically significant (FDR*P* value <0.05).

**Figure 3:**
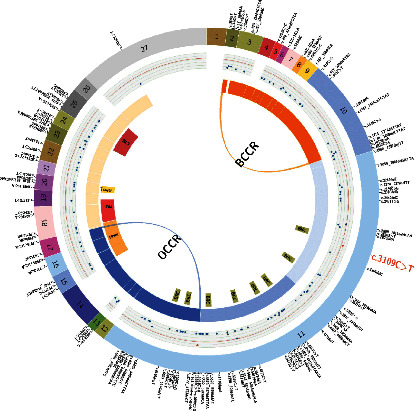
Circos plot representation of all *BRCA2* deleterious mutations identified in this study. The outmost ring displays the 27 exons of *BRCA2* and each variant at corresponding positions. Recurrent variants are highlighted in a red and enlarged font. The second circle is the mutation density graph, each dot corresponds to a variant, and its distance to the outmost ring represents the frequency (the closer the higher; the most prevalent mutation is colored in red). The ROR is represented by the heatmap in the intermediate ring. The next circle displays the *BRCA2* functional domains. The innermost arcs represent previously reported Caucasian-based OCCR/BCCRs. The areas enclosed by the arches indicate the estimated Chinese OCCR/BCCR, which are statistically significant (FDR*P* value <0.05).

A total of 236 deleterious germline mutations in *BRCA1* (Figure 2) and 122 in *BRCA2* (Figure 3) were detected and verified in this study. Several recurrent mutations are identified (Figures 2 and 3 highlighted with red, bigger font). *BRCA1*: c.5470_5477delATTGGGCA (Ile1824AspfsTer3) was the top hit, accounting for 9.5% (50/527) *BRCA1* carriers; the second hit for *BRCA1* was c.981_982delAT, with 4.0% (21/527) prevalence in *BRCA1* carriers; next were c.3770_3771delAG (12/527, 2.3% of *BRCA1* carriers), c.5521delA (11/527, 2.1%), and c.4801A > T (8/527, 1.5%). The *BRCA2*: c.5722_5723delCT was the top hit for *BRCA2* (10/202, 5.0%), followed by *BRCA2*: c.3109C > T (9/202, 4.5%).

### 3.1. Estimated Chinese OCCR/BCCR in BRCA1

We predicted an OCCR at c.1154–c.2111 (Table 2; Figure 2, blue arch) with a relative decrease in breast cancer risk and a relative increase in ovarian cancer risk (ROR = 0.29; 95% CI = 0.15–0.57; FDR-corrected *P* value = 1.2 × 10^−3^). The putative OCCR lies within the largest exon (Exon 10) of *BRCA1* and is partially overlapped with previously reported OCCR (c.1380–c.4062) [8] (Figure 2, blue arc). The putative OCCR entirely or partially spans several functional domains, including the binding sites for Rb, Rad50, c-Myc, and the nuclear localization sequence (NLS) [22]. A putative BCCR was found at c.5470–c.5524 near the 3' end of the CDS (Table 2; Figure 2, orange arch), explained by a relative decrease in ovarian cancer risk (ROR = 3.12, 95% CI = 1.56–5.88, FDR-corrected *P* = 1.2 × 10^−3^). The BCCR lies within the second BRCT domain (c.5268–c.5526) and is also partially overlapped with previously reported BCCR (c.5261–c.5563) [8] (Figure 2, orange arc).

### 3.2. Estimated Chinese OCCR/BCCR in BRCA2

We predicted an OCCR at c.5745–c.7805 + 1 (Table 2; Figure 3, blue arch) with a relative decrease in breast cancer risk and a relative increase in ovarian cancer risk (ROR = 0.38; 95% CI = 0.16–0.86; FDR-corrected *P* value = 0.044). The putative OCCR is partially overlapped with previously reported OCCR (c.6645–c.7471) [8] (Figure 3, blue arc), spanning from the last two BRC repeats within exon 11 (the largest exon of *BRCA2*) to approximately the boundary of exons 17 and 18, which contains the most part of the helical DNA-binding domain (c.7437–c.8001). A putative BCCR was found at c.10–c.2176 (Table 2; Figure 3, orange arch), explained by a relative increase in breast cancer risk and a relative decrease in ovarian cancer risk (ROR = 3.47, 95% CI = 1.51–7.96, FDR-corrected *P*=0.012); it is also partially overlapped with previously reported BCCR (c.1–c.596, c.772–c.1806) [8] (Figure 3, orange arcs). The putative BCCR spans from the 5' end of the CDS to approximately the boundary of exons 10 and 11.

### 3.3. Estimated Penetrance of Chinese Founder Mutations Greatly Differ

Of the six founder mutations examined, the two most prevalent mutations (*BRCA1*: c.5470_5477del and *BRCA1*: c.981_982del) were estimated to have penetrance lower than 0.35; one (*BRCA2*: c.3109C > T) with either above or lower than 0.35 penetrance subjected to family history (high penetrance only occurs in carriers with positive family history), and three with small sample sizes (*BRCA1*: c.3342_3345del, *BRCA1*: c.5154G > A, and *BRCA1*: c.4801A > T) showed complete penetrance (100%) due to no carriers found in control. Our results demonstrated that the penetrance of different *BRCA1/2* deleterious mutations greatly varies and shows a large deviation from the expected value.

### 3.4. The Founder BRCA1: c.5470_5477del Is Estimated to Have Emerged More than 2000 Years Ago

**Figure 4:**
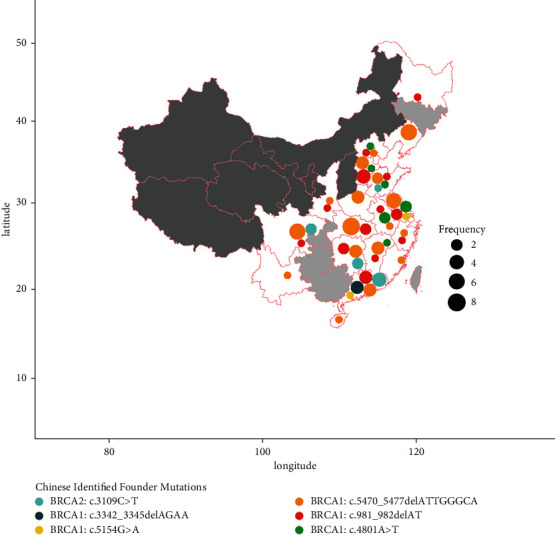
Geographical distribution of the six known Chinese founder mutations. Areas with sample size <100 are colored in light gray; areas with sample size <50 are colored in dark gray. The total sample size is 7,919. Spot size (defined as the spot size unit is based on the number count of the corresponding mutation carriers) indicates the frequency of mutation, and spot color indicates different founder mutations.

As mentioned above, the *BRCA1*: c.5470_5477del was the most recurrent deleterious mutation, accounting for 9.5% of *BRCA1* mutation carriers. Haplotype analysis was carried out on 31 unrelated patients and 50 unrelated controls without the mutation. The haplotype analysis was performed independent of the work of Meng et al. [6]; similar to their findings, our haplotype analysis suggested a strong founder effect (Supplementary Table S1) of the mutation. Moreover, carriers of this variant are distributed throughout the country (Figure 4), except provinces with sampling size <100 (regions colored in gray; total sample size: 7,919). Compared with other known Chinese founder mutations, BRCA1: c.5470_5477del (orange dots) showed the highest allele frequency and the most thorough spread in terms of geographical location, indicating a relatively early emergence of the mutation. We used DMLE + 2.3 to estimate the distribution of possible mutation age (years) under each growth rate (Supplementary Figure S2), and then, we compared the estimated mutation age (peak) and the actual time from which the growth rate is drawn. Among the seven growth rates used, the estimated mutation age ($\hat{n}$) and the actual time (*n*) showed the best correlation with 1.54‰ (2 A.D-present), for which $\hat{n}$ = 2,090 and *n* = 2,018. The estimated time (∼2,090 years ago, i.e., ∼70 B.C.) of the emergence of the mutation lies within the period of the Han dynasty (206 B.C–220 A.D.), currently known as the second imperial dynasty of China.

## 4. Discussion

We have presented a large-scale *BRCA1/2* screening in both cancer patients and cancer-free individuals throughout China. We preliminarily defined the Chinese OCCR/BCCRs in *BRCA1/2* and compared them with the previously reported OCCR/BCCRs based on mainly Caucasian population. We estimated penetrance for each of the six known Chinese founder mutations and demonstrated great variations between them. Unlike previous studies that were based on patients and/or their family members, this study included a large number of samples from the normal population, allowing us to assess the risk of a mutation carrier without a family history, which can significantly differ from the risk of those with family history. We also performed haplotype analysis on the most recurrent founder mutation *BRCA1*: c.5470_5477del and estimated its time of emergence to be ∼2,090 years ago within the Han dynasty.

Our OCCR/BCCRs partially overlap with previously reported OCCR/BCCRs drawn from the White/Jewish (Ashkenazi) population (Figures 2 and 3) [8]. We observed both in the Chinese and the White/Jewish studies the OCCRs' position at the middle of the gene where the largest exon with the highest mutation rate is located and with binding sites for key proteins involved in DNA repair processes such as *RAD51* and *PALB2* [22, 23], whereas BCCRs tend to position at the ends (5' and/or 3') of the gene where smaller exons with secondary peaks of mutation rate are located and include transcription activation domain (TAD) and DNA-binding domain (DBD). OCCRs tend to be longer than BCCRs. The location of OCCR/BCCRs is thought to be mainly determined by two factors: the biological impact caused by mutations within a certain region and the frequency of these mutations detected within the studied population. While the former is likely not to be affected by ethnicity and may explain the concordant parts of the OCCR/BCCRs, the latter is known to be considerably affected by ethnicity and may explain the parts with discordance (38% of Chinese *BRCA1/2* variants have not been reported in other populations [24]). Indeed, the Caucasian OCCR/BCCRs and Chinese OCCR/BCCRs show concordance with hotspot exons (i.e., frequently mutated exons) drawn from BIC and dbBRCA-Chinese databases [24], respectively. The difference in mutation frequency of each exon between the two databases may explain some of the differences shown between our Chinese OCCR/BCCRs and the previously reported Caucasian-based OCCR/BCCRs. For example, the reported mutation rate of *BRCA1* Exon5 in BIC is threefold of that in the dbBRCA-Chinese database, which may explain the presence of a BCCR in Caucasian data and its absence in our Chinese data (note that due to historical reasons, the first exon was named exon 2 so that exon 11 is the largest exon, as seen in many literature and databases and that exons 2, 5, and 11 are equal to exons 1, 4, and 10 in our study). However, the exact mechanism of how the biological impact is affected by the position of the mutation is unclear, since the vast majority of *BRCA2* mutations are truncating and able to trigger nonsense-mediated mRNA decay (NMD) [25]; truncated *BRCA2* proteins are cytoplasmic [26] and unable to enter the nucleus due to lack of an intact nuclear localization signal. One possible explanation might be that NMD is not removing all truncating transcripts, which translate into truncated proteins and might compete with intact proteins for binding partners at the cytoplasm, or even become able to enter the nucleus through the carriage by a binding partner.

With the awareness of the great heterogeneity in cancer risks for mutations in different regions as evidenced by the OCCR/BCCRs, we focused on estimating independent penetrance for each recurrent mutation instead of a single penetrance for the whole gene. The two most prevalent mutations, which have been shown to spread through the country, are estimated to have relatively low penetrance, while those less prevalent ones residing in a local scope tend to have higher or complete penetrance. Our results demonstrated the “one risk does not fit all” as previously suggested by De Bock et al. [27]. Not every BRCA1/2 carrier has the same risk of developing cancers; some may never develop cancer throughout their lives. It is, therefore, important to separately consider when assessing cancer risks for BRCA1/2 carriers with different genotypes. For carriers of low-risk genotypes, regular follow-up surveillance is required. Follow-up screening for breast tumors is based on clinical palpation combined with imaging examination (MRI examination is preferred if available or ultrasound combined with mammography), while screening for ovarian cancer includes CA125 examination and ultrasound examination [28]. A more preventive strategy such as risk-reducing resection should be considered for carriers with high-risk genotype [29]. In BRCA mutation carriers, risk-reducing salpingo-oophorectomy (RRSO) alone or combined with bilateral risk-reducing mastectomy (BRRM) significantly reduces all-cause mortality, risk of ovarian cancer, and risk of breast cancer [30, 31]. When is the optimal time to perform RRSO? It is recommended to be performed at the age of 35–40 years after completion of childbearing for those with the BRCA1 mutation and at the age of 40–45 years for those with BRCA2 mutation [32]. In addition, complications associated with prophylactic surgical resection should not be ignored. Among them, the occurrence of early menopause is the most feared by both patient and physician after RRSO. Therefore, before RRSO is performed, patients should be informed of the common sequelae of medically induced menopause and informed of the benefits and risks of the appropriate remedies. Recent studies have found that HRT seems to be a safe therapeutic option in BRCA 1 and BRCA 2 mutation carriers undergoing RRSO and that the risk of BC treated with HRT after receiving RRSO was not significantly elevated [33]. Furthermore, assisted reproductive technology [34] should be considered for carriers who are of childbearing age and with high-risk genotype to prevent the passage of the high-risk allele. Women at high risk have a relatively short window of “reproductive age” and if they have not yet completed childbearing and have to consider RRSO, assisted reproduction with oocyte and/or embryo cryopreservation is recommended [35]. Therefore, the management of high-risk women with BRCA germline mutations requires the multidisciplinary involvement of geneticists, gynecologic oncologists, breast surgeons, and psychologists. For those putative high-risk mutations that seemed to be limited within a relatively small local area, it is necessary to carry out concentrated screening in order to further reveal the true penetrance in a larger sample size with standard incidence ratio (SIR), and if the high penetrance is confirmed, to identify more carriers of the mutation and take early preventative measures.

We estimated the time of emergence of the currently most recurrent Chinese founder mutation (also the most widely spread and probably the oldest), *BRCA1*: c.5470_5477del. Our estimation indicated that the emergence of this mutation may have happened ∼2,090 years ago (∼70 B.C.) during the Han dynasty, or more specifically, during the Western Han (Xi Han, 206 B.C.-8 A.D.). The thorough spread of the mutant allele throughout the country is most likely to be explained by multiple large-scale population migration events caused by frequent wars. There have been three major waves of population migration in Chinese history: the first started from the end of the Han dynasty (220 A.D.), i.e., the start of the Three Kingdoms period when the country was divided into three and suffered from long-lasting wars, until the end of the Southern and Northern dynasties (also known as the start of the Sui dynasty, 589 A.D.); the second occurred during the An Shi Rebellion in Tang dynasty (755 A.D.); the third happened during the Jingkang Incident (1127 A.D.), which has led to the end of the Northern Song dynasty. The three major migrations all happened after the estimated time of emergence of *BRCA1*: c.5470_5477del. There are 290 years (∼14.5 generation) between the estimated emergence (70 B.C.) and the first wave of migration (220 A.D.), which would be enough for the initial accumulation of mutant alleles so that the founder mutation can survive wars and natural disasters and inherit for more than two thousand years.

There are several limitations of this study. It is retrospective, and the number of subjects may be insufficient for a comprehensive estimation of the 1.4 billion Chinese population; follow-up time for *BRCA1/2* carriers is short; most of the cancer-free carriers are under 70 years old, which can cause underestimation of the cancer risk; the portion of *BRCA1/2* carriers in cancer-free individuals is higher than expected, which is likely due to the fact that those with family histories are more willing to participate in testing; we did not include large rearrangement events of *BRCA1/2* in our research, which may account for more than 5% of all *BRCA1/2* mutations [36]; due to the lack of genome-wide single-nucleotide polymorphism of all subjects included, we have no access to the population stratification considering the geographical difference and 56 ethnic origins of the Chinese population.

## 5. Conclusions

We preliminarily defined the OCCR/BCCRs based on a large number of Chinese samples. The Chinese OCCR/BCCRs partially overlap with the previously defined OCCR/BCCRs based on Caucasian samples. We estimated the penetrance of the six major Chinese founder mutations, respectively, and demonstrated great variations between them, which strongly suggests that cancer risks should be calculated and separately considered depending on the genotype rather than looking at a fixed risk figure. From a rigorous perspective, it is still necessary to calculate the true penetrance in a larger study cohort with population-based standardized incidences of breast and ovarian cancers. Finally, we investigated the most prevalent and nationally spread Chinese founder mutation *BRCA1*: c.5470_5477del and estimated that it has more than two thousand years of history.

## Figures and Tables

**Table 1:** Clinical characteristics and germline *BRCA1/2* status of the samples in this study.

Cancer site	Number, *n* = 7919 (%)	Median age (years)	*P* value	BRCA1/2 carriers, *n* = 729 (%)	Non-carriers, *n* = 7190	*P* value
Breast	2400 (30.31%)	45.00 (38.00–52.00)	<0.0001	285 (11.88%)	2115 (88.13%)	0.041
Ovary	1697 (21.43%)	52.00 (46.00–59.00)		332 (19.32%)	1365 (80.68%)	
Breast and ovary	32 (0.40%)	55.00 (48.25–60.75)		16 (48.48%)	16 (51.52%)	
Breast or ovary and other sites	33 (0.42%)	53.50 (45.00–61.00)		1 (3.03%)	32 (96.97%)	
Other sites	116 (1.46%)	61.00 (49.25–70.75)		5 (4.31%)	111 (95.69%)	
Cancer-free	3641 (45.98%)	36.00 (30.00–43.00)		90 (2.47%)	3551 (97.53%)	

**Table 2:** Ovarian cancer cluster regions (OCCR) and breast cancer cluster regions (BCCR) in *BRCA1* and *BRCA2*.

Gene	#Bin	No. of carriers	Putative region	Bin-starting nucleotide	Bin-ending nucleotide	Ratio (OR-breast: OR-ovarian)	*P* value	FDR *P* value
BRCA1	1	58		34	335	0.81 (0.45–1.47)	0.49	0.63
	2	58		397	1115	0.78 (0.44–1.41)	0.4126	0.6189
	**3**	**63**	**OCCR1**	**1154**	**2111**	**0.29 (0.15–0.57)**	**0.0001539**	**0.0012186**
	4	58		2127	3229	1.07 (0.61–1.89)	0.8066	0.8066
	5	62		3257	3771	0.78 (0.44–1.37)	0.3852	0.6189
	6	59		3841	4573	0.88 (0.48–1.60)	0.6704	0.7542
	7	58		4609	5095	1.65 (0.92–2.97)	0.09114	0.27342
	8	59		5096	5468-1	1.46 (0.80–2.66)	0.2158	0.48555
	**9**	**66**	**BCCR1**	**5470**	**5524**	**3.12 (1.65–5.88)**	**0.0002708**	**0.0012186**

BRCA2	**1**	**41**	**BCCR2**	**10**	**2176**	**3.47 (1.51–7.96)**	**0.002485**	**0.012425**
	2	41		2244	4038	1.00 (0.47–2.14)	0.9981	0.9981
	3	48		4151	5723	0.49 (0.23–1.04)	0.0603	0.1005
	**4**	**41**	**OCCR2**	**5745**	**7805 + 1**	**0.38 (0.16–0.86)**	**0.01789**	**0.044725**
	5	40		7835	10150	1.63 (0.73–3.62)	0.2281	0.285125

OR: odds ratio; FDR: false discovery rate.

**Table 3:** Age estimation analysis using population data from different historical time points.

Time point	Population (thousand)	Average growth rate (‰)	Time till now (yrs)	Estimated mutation age (yrs)
684 B.C.	11,840	1.76	2,704	2,145
2 A.D.	59,590	1.54‰	2,018	2,090
609 A.D.	46,020	2.40‰	1,411	1,534
1110 A.D.	46,730	3.71‰	910	1,585
1403 A.D.	66,600	4.87‰	617	992
1741 A.D.	143,410	7.81‰	279	602
1949 A.D.	541.67	6.50‰	71	1,006

## Data Availability

The data that support the findings of this study are available on request from the corresponding author. The data are not publicly available due to privacy or ethical restrictions.

## Additional Points

This manuscript has previously been released as a preprint at https://www.medrxiv.org/content/10.1101/2020.07.20.20135202v1 [37].

## Supplementary Materials

Supplementary Materials Table S1: most likely haplotype analysis of BRCA1 c.5470 5477delATTGGGCA mutation patient carriers. Figure S1: genetic map of the nine STR markers used in the haplotype analysis of BRCA1: c.5470_5477del (p.Ile1824AspfsTer3). Figure S2: age estimation of BRCA1: c.5470_5477del. Seven different growth rates (gr, %) were used together with the haplotype profiles as input to DMLE + 2.3, and a distribution of mutation age vs probability (frequency) was estimated for each simulation. The highest probability was achieved at gr = 0.154%, for which the estimated mutation age was 2090 years.
