# Supplementary material for: Assessing the Variations in Breast/Ovarian Cancer Risk for Chinese BRCA1/2 Carriers
Source: J Oncol. 2022 Mar 26;2022:9390539. doi: 10.1155/2022/9390539 (PMC8976609; doi:10.1155/2022/9390539)
Supplement: Supplementary Materials — Table S1: most likely haplotype analysis of BRCA1 c.5470 5477delATTGGGCA mutation patient carriers. Figure S1: genetic map of the nine STR markers used in the haplotype analysis of BRCA1: c.5470_5477del (p.Ile1824AspfsTer3). Figure S2: age estimation of BRCA1: c.5470_5477del. Seven different growth rates (gr, %) were used together with the haplotype profiles as input to DMLE + 2.3, and a distribution of mutation age vs probability (frequency) was estimated for each simulation. The highest probability was achieved at gr = 0.154%, for which the estimated mutation age was 2090 years. [file 9390539.f1.zip › 9390539.f1/Figure S1.pdf]

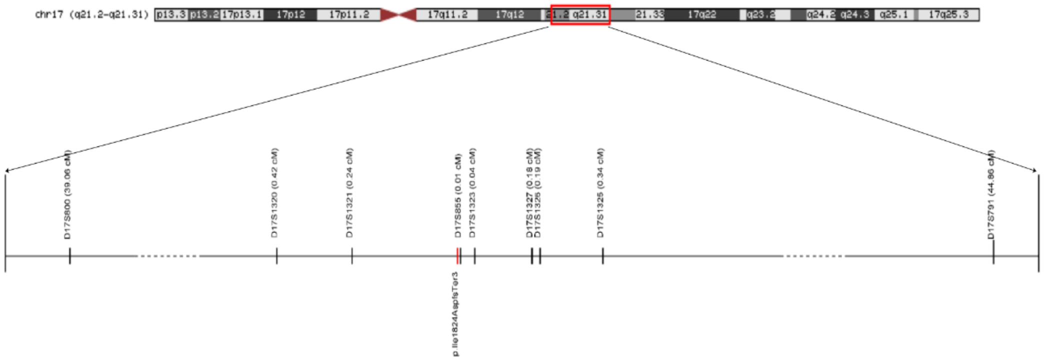

Figure S1. Genetic map of the nine STR markers used in the haplotype analysis of BRCA1:c.5470_5477del (p.Ile1824AspfsTer3).
