# Supplementary material for: Assessing the Variations in Breast/Ovarian Cancer Risk for Chinese BRCA1/2 Carriers
Source: J Oncol. 2022 Mar 26;2022:9390539. doi: 10.1155/2022/9390539 (PMC8976609; doi:10.1155/2022/9390539)
Supplement: Supplementary Materials — Table S1: most likely haplotype analysis of BRCA1 c.5470 5477delATTGGGCA mutation patient carriers. Figure S1: genetic map of the nine STR markers used in the haplotype analysis of BRCA1: c.5470_5477del (p.Ile1824AspfsTer3). Figure S2: age estimation of BRCA1: c.5470_5477del. Seven different growth rates (gr, %) were used together with the haplotype profiles as input to DMLE + 2.3, and a distribution of mutation age vs probability (frequency) was estimated for each simulation. The highest probability was achieved at gr = 0.154%, for which the estimated mutation age was 2090 years. [file 9390539.f1.zip › 9390539.f1/Table S1.docx]

|  | **Table S1. Most likely haplotype analysis of BRCA1 c.5470_5477delATTGGGCA mutation patients carriers** | | | | | | | | | |
| --- | --- | --- | --- | --- | --- | --- | --- | --- | --- | --- |
|  | **Sample** | **D17S800** | **D17S1320** | **D17S1321** | **D17S855** | **D17S1323** | **D17S1327** | **D17S1326** | **D17S1325** | **D17S791** |
|  | P01 | 166 | 171 | 151 | 147 | 151 | 129 | 105 | 201 | 161 |
|  | P02 | 166 | 171 | 151 | 147 | 151 | 129 | 105 | 201 | 161 |
|  | P03 | 166 | 171 | 151 | 147 | 151 | 129 | 105 | 201 | 161 |
|  | P04 | 166 | 171 | 151 | 147 | 151 | 129 | 105 | 201 | 161 |
|  | P05 | 166 | 171 | 151 | 147 | 151 | 129 | 105 | 201 | 169 |
|  | P06 | 166 | 171 | 151 | 147 | 151 | 129 | 105 | 201 | 174 |
|  | P07 | 166 | 171 | 151 | 147 | 151 | 129 | 105 | 197 | 161 |
|  | P08 | 166 | 171 | 151 | 147 | 151 | 129 | 105 | 202 | 161 |
|  | P09 | 166 | 171 | 151 | 147 | 150 | 129 | 105 | 201 | 161 |
|  | P10 | 166 | 171 | 151 | 149 | 151 | 129 | 105 | 201 | 161 |
|  | P11 | 166 | 171 | 150 | 147 | 151 | 129 | 105 | 201 | 161 |
|  | P12 | 166 | 171 | 150 | 147 | 151 | 129 | 105 | 201 | 161 |
|  | P13 | 166 | 171 | 148 | 147 | 151 | 129 | 105 | 201 | 161 |
|  | P14 | 166 | 171 | 151 | 147 | 151 | 129 | 105 | 199 | 180 |
|  | P15 | 166 | 171 | 151 | 147 | 151 | 129 | 103 | 201 | 175 |
|  | P16 | 166 | 171 | 151 | 147 | 151 | 129 | 103 | 199 | 161 |
|  | P17 | 166 | 171 | 150 | 147 | 151 | 129 | 105 | 201 | 174 |
|  | P18 | 166 | 171 | 149 | 147 | 151 | 129 | 105 | 201 | 176 |
|  | P19 | 166 | 171 | 152 | 147 | 151 | 129 | 105 | 201 | 174 |
|  | P20 | 166 | 171 | 150 | 147 | 151 | 129 | 105 | 201 | 174 |
|  | P21 | 166 | 171 | 150 | 147 | 151 | 129 | 105 | 201 | 174 |
|  | P22 | 166 | 171 | 150 | 147 | 151 | 129 | 105 | 193 | 161 |
|  | P23 | 166 | 171 | 150 | 147 | 151 | 128 | 105 | 201 | 161 |
|  | P24 | 170 | 171 | 151 | 144 | 151 | 129 | 105 | 201 | 161 |
|  | P25 | 170 | 171 | 150 | 147 | 151 | 129 | 105 | 201 | 161 |
|  | P26 | 166 | 171 | 151 | 147 | 151 | 129 | 103 | 199 | 176 |
|  | P27 | 166 | 171 | 151 | 147 | 150 | 129 | 104 | 201 | 174 |
|  | P28 | 170 | 171 | 151 | 147 | 150 | 128 | 105 | 201 | 161 |
|  | P29 | 166 | 171 | 151 | 145 | 151 | 129 | 105 | 199 | 186 |
|  | P30 | 166 | 171 | 150 | 147 | 151 | 129 | 103 | 199 | 182 |
|  | P31 | 166 | 171 | 149 | 147 | 151 | 128 | 103 | 201 | 177 |
|  | **freq_control** | 0.51 | 0.55 | 0.1 | 0.34 | 0.52 | 0.61 | 0.09 | 0.01 | 0.25 |
|  | **freq_case** | 0.726 | 0.823 | 0.306 | 0.71 | 0.774 | 0.806 | 0.468 | 0.468 | 0.29 |
|  | **P-value** | 0.01069 | 0.0007361 | 0.002219 | 0.00001009 | 0.002147 | 0.01458 | 1.014E-07 | 1.422E-12 | 0.7025 |
|  | **Mb** | 2.141551 | 0.417813 | 0.244314 | 0.006935 | 0.040235 | 0.177651 | 0.187296 | 0.337722 | 3.658456 |
|  | The numbers equal to the amplicon size containing the STR markers. The parts of the likely ancestral haplotype shared by all patients are indicated in shades. freq_case/control: frequency of the allele shared by the patients and the control sample; Mb: Physical distance in Mb obtained from UCSC Genome Browser. | | | | | | | | | |
